# Ultrastructural Localization of Polygalacturonase in Ethylene-Stimulated Abscission of Tomato Pedicel Explants

**DOI:** 10.1155/2014/389896

**Published:** 2014-03-24

**Authors:** Ming-Fang Qi, Tao Xu, Wei-Zhi Chen, Tian-lai Li

**Affiliations:** ^1^Department of Horticulture, Key Laboratory of Protected Horticulture, Ministry of Education, No. 120 Donglin Road, Shenyang City, Liaoning 110866, China; ^2^Shenyang Agricultural University, No. 120 Donglin Road, Shenyang City, Liaoning 110866, China

## Abstract

Polygalacturonase (PG) is crucial in plant organ abscission process. This paper investigated the cellular and subcellular localization of PG in ethylene-stimulated abscission of tomato pedicel explants. Confocal laser scanning microscopy of abscission zone sections with the fluorescent probe Cy3 revealed that PG was initially accumulated in parenchyma cells in cortical and vascular tissues after 8 h of ethylene treatment and then extended throughout the abscission zone when the abscission zone separated at 24 h after ethylene treatment. At the subcellular level, transmission electron microscopy with immunogold staining showed that PG showed abundant accumulation in the cortical and vascular tissues at 8 h after ethylene treatment, and the distribution area extended to the central parenchyma cells at 16 h after ethylene treatment. In addition, PGs were observed in the distal and proximal parts of the tomato pedicel explants throughout the abscission process. The results provided a visualized distribution of PG in the pedicel abscission zone and proved that PG was closely related to abscission.

## 1. Introduction

Abscission is the process in which organs are separated from the parent plant; this process involves multiple changes in cell structure, metabolism, and gene expression [1]. Abscission usually occurs in the abscission zone of plants. The abscission zone contains small, square-shaped cells with a dense cytoplasm [2, 3]. Abscission has three classes, namely, normal abscission (such as abscission of ripened fruit and seed), metabolic abscission due to the completion of reproductive and vegetative growth (such as premature shedding of fruit and unpollinated flowers), and abscission due to environmental stresses (such as heat, cold, and light). The development and mechanisms of plant hormones, such as the interplay of ethylene and auxin, are well documented. However, detailed mechanisms on the regulation and control of these processes remain unknown.

Abscission signals, including environmental and developmental cues, can be sensed by the cells in the abscission zone. These signals initiate abscission. Plant hormones, especially ethylene and auxin, have important functions in abscission. Several studies have modeled the regulation of ethylene and auxin [1, 2, 4, 5]. Ethylene accelerates abscission, whereas auxin restricts the process.

Studies have proven that cell wall hydrolases, such as *β*-1,4-glucanase, polygalacturonase (PG), and chitinase, have important functions after the initiation of abscission [3, 6–8]. Particularly, PGs are essential for the degradation of pectin, a crucial component of the adhesive material between cells. Gene expression profiling studies have shown that increased PG expression in abscission is closely related to flower abscission [7–11]. However, the location of PG protein in the abscission zone remains unclear. A recent study in tomato has suggested that enzyme expression and synthesis within abscission zone cells are asymmetric with regard to the proximal-distal axis [12]. Tomato flower pedicel is an ideal material to study abscission because of its prominent abscission zone. The current study aims to locate PGs in the tomato abscission zone during ethylene-induced abscission using immunogold staining.

## 2. Materials and Methods

### 2.1. Plant Material and Ethylene Treatment

Liaoyuanduoli, an indeterminate and popular tomato (*Lycopersicon esculentum* Mill.) variety in Northeast China, was used and cultured as previously mentioned [13, 14]. The flowers were collected when all the petals opened. The opening angle was 90° or more, and the color of the petals was fresh yellow. Floral explants were excised from the inflorescence and immediately used for experiments.

### 2.2. Ethylene Treatment

Two transparent glass containers were used for ethylene treatment [14]. Pure ethylene (purity: 99.99%, from Shenyang Beidu Special Gas Co., Ltd.) was injected into the container, and the ethylene concentration was maintained at 20 *μ*L L^−1^. After 0, 8, 16, and 24 h, the relative abscission rate was investigated, and the abscission zone was excised to locate PGs. Forty explants served as one replicate.

### 2.3. Analysis of PG Activity

PG activity was measured according to the method of Wang et al. [14]. One unit of activity was defined as a 1% loss in the reaction solution viscosity per 1 h per 1 g of fresh tissue.

### 2.4. PG Antibody Production

We obtained a PG antibody from Abmart (Shanghai, China) using New Zealand white rabbits according to the published sequences of TAPG 1 (53–64) in NCBI:C-NKPSTINVPIGK-NH2 [13].

### 2.5. Frozen Section and Immunofluorescence Staining

Tomato pedicel abscission zones (approximately 3 mm) were cut using a double-sided blade and then fixed with formaldehyde and glutaraldehyde (4% formaldehyde : 0.5% glutaraldehyde = 1 : 1) for 1 h at 4°C. The abscission zone was embedded in optimal cutting temperature compound, frozen, and then sectioned to 50 *μ*m thickness with a Leica CM1850 (Germany) frozen section machine.

Afterward, the frozen section was blocked with 2% bovine serum albumin for 30 min at 37°C, and excess serum around the tissue was removed. The section was added with PG antibody (used at a dilution 1 : 50) and then incubated at 37°C overnight. The slides were rinsed thoroughly with phosphate-buffered saline (PBS), and excess PBS around the tissue was removed. Subsequently, the sample was added with anti-rabbit Cy3-conjugated secondary antibody (Wuhan Boster Bio-engineering Limited Company, China) and then incubated for 30 min at 37°C. The sample was rinsed thoroughly with PBS and then mounted with glycerin. Observations were conducted with a confocal laser scanning microscope (ZEISS, LSM) at excitation and emission wavelengths of 554 and 570 nm, respectively.

### 2.6. Ultrathin Sections and Immunogold Staining

Tomato pedicel abscission zones (approximately 3 mm) were cut using a double-sided blade and then fixed in a PBS mixture of formaldehyde and glutaraldehyde (4% formaldehyde : 0.5% glutaraldehyde = 1 : 1) for 3 h at 4°C. Osmium tetroxide was subsequently added, and fixing was continued for 1 h.

After fixing, the tissue materials were washed in 0.1 M PBS (pH 7.4) and then dehydrated through 70%, 80%, 90%, and 95% ethanol (30 min per step). The sections were infiltrated for 1 h with 1 : 1 LR white acrylic resin, 100% ethanol, and then in pure LR white for 24 h. Final embedding was conducted in gelatin capsules, followed by polymerization in an oven at 60°C for 36 h.

After complete polymerization, the abscission zone tissues were obtained and made into ultrathin sections (50 nm). The sections were collected on formvar-carbon-coated nickel grids and allowed to dry overnight prior to staining.

The sections were rinsed twice (2 min each) with PBS-Tween 20. After being blocked in 2% bovine serum albumin for 30 min at 37°C, slide-mounted sections were covered in a droplet of primary antibody and incubated overnight at 4°C.

The primary antibody against PG and the immunogold reagent were diluted to 1 : 50 and 1 : 100 (15 nm, Bio-Rad Laboratories, USA), respectively, with 50 mM phosphate-buffered saline at pH 7.2.

The sections were rinsed three times (2 min each) with PBS-Tween20 and then incubated with the immunogold reagent at 37°C for 30 min. The sample was rinsed thrice (2 min each) with PBS-Tween20 and then with distilled water before it was allowed to dry at room temperature.

The treated sections were observed and photomicrographed with a transmission electron microscope (JEM100CX-II, Japan).

## 3. Results

### 3.1. Effects of Ethylene on Pedicel Explant Abscission and PG Activity

**Figure 1:**
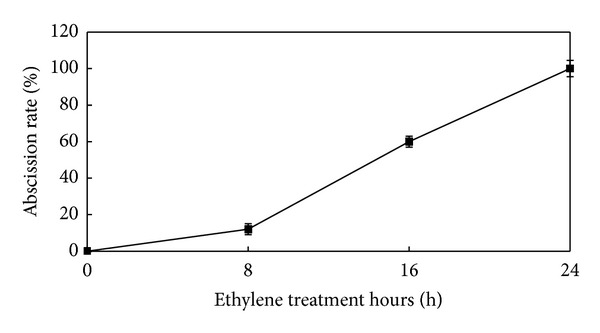
Effect of ethylene on abscission rate of tomato pedicel explants. Vertical bars indicate ± S.E. (*n* = 3).

**Figure 2:**
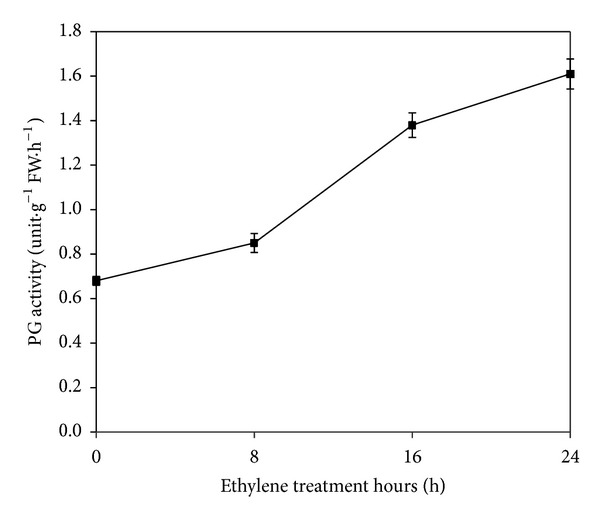
Effect of ethylene on polygalacturonase (PG) activity in tomato pedicel explants. Vertical bars indicate ± S.E. (*n* = 3).

The abscission rate and PG activity of the tomato pedicel explants treated with ethylene were investigated throughout the 24 h treatment (Figures 1 and 2). A few pedicel explants separated from the plant, the abscission rate rapidly increased, and 100% abscission rate was reached at 8, 16, and 24 h after ethylene treatment, respectively. Similar to the abscission rate, PG activity was relatively low at 0 and 8 h after ethylene treatment but increased rapidly at 16 and 24 h after the treatment. These results suggested that PG activity and abscission rate were closely related.

### 3.2. Cellular Location of PG in the Abscission Zone during Ethylene-Induced Abscission

**Figure 3:**
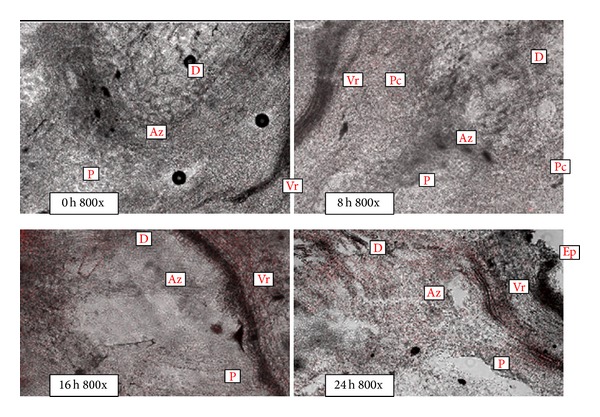
Immunolocalization of polygalacturonase (PG) in tomato pedicel with Cy3 fluorescent labeling. D, Az, and P indicate the distal, abscission zone, and proximal side, respectively. Pc: parenchyma cells; Ep: epidermis; Vr: vascular bundle region.

PG activity is closely correlated with abscission. Thus, understanding the function of PG in abscission would be helpful in locating PG in the abscission zone. Thus, we observed the distribution of PG in the abscission zone during ethylene-stimulated abscission using a fluorescent antibody-Cy3-conjugated secondary antibody (Figure 3). Results showed that Cy3 fluorescent labeling of the abscission zone containing dense small cells was very weak at the beginning of ethylene treatment. At 8 h after ethylene treatment, the fluorescent labeling strengthened throughout the abscission zone, especially in the parenchyma cells around the vascular tissue. The fluorescent level further increased at 16 h after the treatment. When the abscission layer was formed at 24 h after ethylene treatment, the distribution of fluorescent labeling presented an even higher level at the abscission zone.

### 3.3. Subcellular Location of PG in the Abscission Zone during Ethylene-Induced Abscission

**Figure 4:**
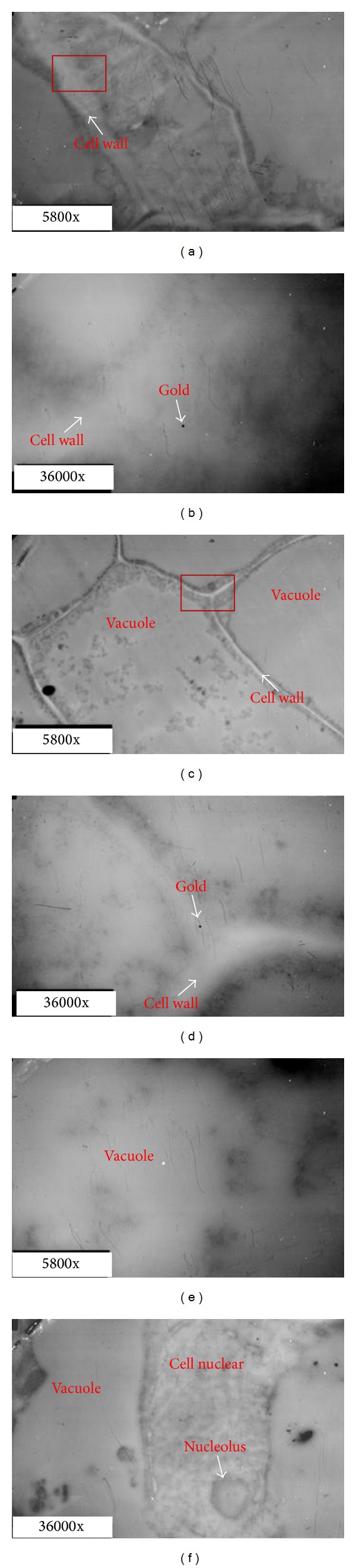
Transmission electron micrographs of tomato pedicel and localization of polygalacturonase (PG) in abscission zone cells with immunogold labeling (0 h). (a) and (b): cortex; (c) and (d): central parenchymatous region; (e): vacuole; (f): cell nucleus; (b) and (d) are enlarged photos for the rectangles region in (a) and (c), respectively.

To find the accurate location of PG in the abscission zone, immunogold staining was further used to fix the position of PG at the cellular level. The results showed that the abscission zone tissue was intact and well structured at 0 h after ethylene treatment (Figure 4). The cortical cells had thick cytoplasm, cell membrane, and cell wall. At this point, a very small amount of gold particles was observed in the cells of the cortex and vascular tissues, in the cell wall, or in the cytoplasm near the cell wall (Figures 4(a) to 4(d)). Gold particles were not observed in the vacuole (Figure 4(e)) and nucleus (Figure 4(f)). These results suggested that PGs were expressed in the cells of the abscission zone but at a low level before the initiation of abscission.

**Figure 5:**
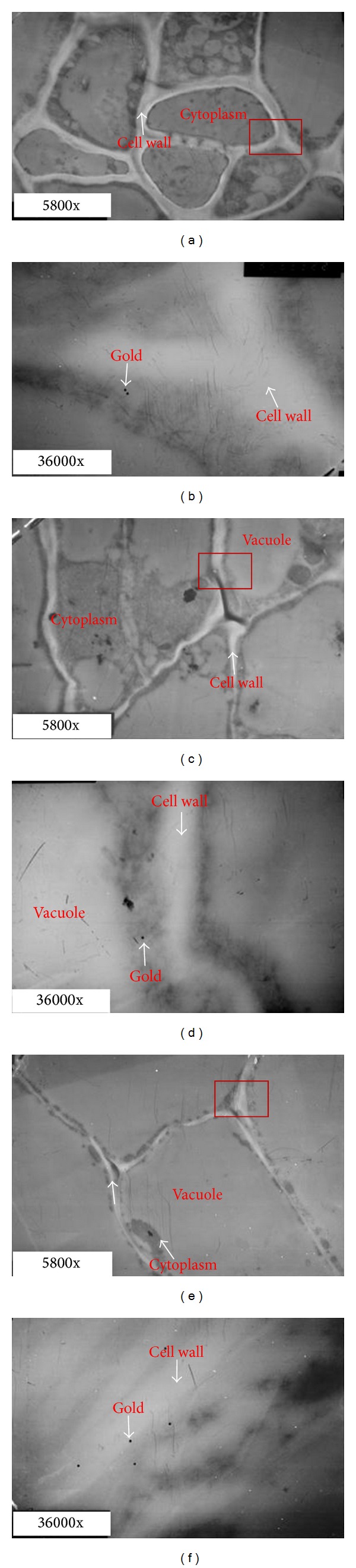
Transmission electron micrographs of tomato pedicel and localization of polygalacturonase (PG) in abscission zone cells by immunogold labeling (8 h). (a) and (b): cortex; (c) and (d): vascular region; (e) and (f): central parenchymatous region (b); (d) and (f) are enlarged photos for the rectangles region in (a), (c), and (e), respectively.

At 8 h after ethylene treatment, the structure of most of the unseparated pedicels remained intact and showed no significant differences (Figure 5). However, the gold particles increased significantly in the parenchyma cells in the cortex (Figure 5(b)) and vascular tissues (Figure 5(d)), as well as in the central parenchyma cells (Figures 5(e) and 5(f)). Similar to the observation at 0 h, most of the gold particles were still near the cell wall.

**Figure 6:**
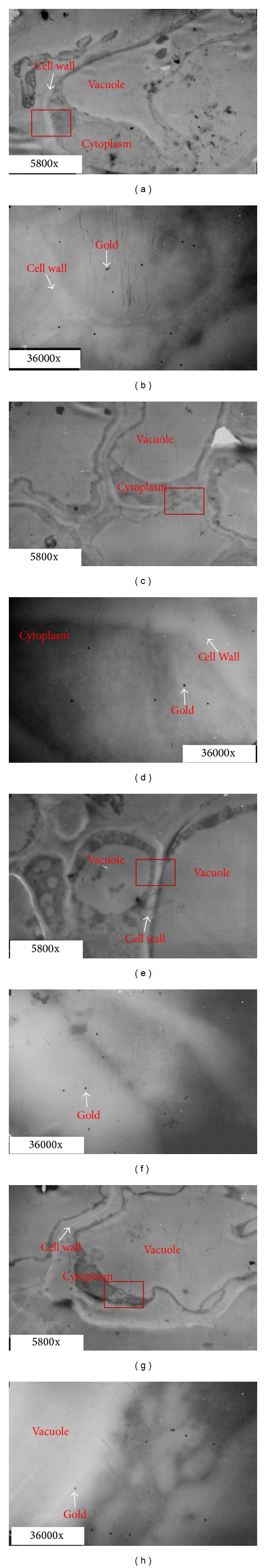
Transmission electron micrographs of tomato pedicel and localization of polygalacturonase (PG) in abscission zone cells by immunogold labeling (16 h). (a) and (b): cortex; (c) and (d): vascular region; (e) and (f): central parenchymatous region; (g) and (h): vacuole; (b), (d), (f),and (h) are enlarged photos for the rectangles region in (a), (c), (e), and (g), respectively.

After 16 h of ethylene treatment (Figure 6), abscission zone tissues started to decompose, and part of the cortical cells began to break down. As a result, the cell structure and cell wall in some areas were destroyed, and plasmolysis began to occur (Figure 6(a)). The structure of the vascular tissue was also damaged, and the cells were deformed (Figure 6(c)). The parenchyma cells in the central region became oval and were about to separate (Figure 6(d)). At this point, a large amount of gold particles were distributed in various parts of the organization, especially in the cortical cells (Figure 6(b)). Most of the gold particles were distributed in the cell wall, cell membrane, and neighboring cytoplasm. Gold particles were also observed in the vacuole (Figures 6(g) and 6(h)). These results suggested that polygalacturonic acid enzymes were involved in cell wall degradation and that their expression was closely related to abscission.

**Figure 7:**
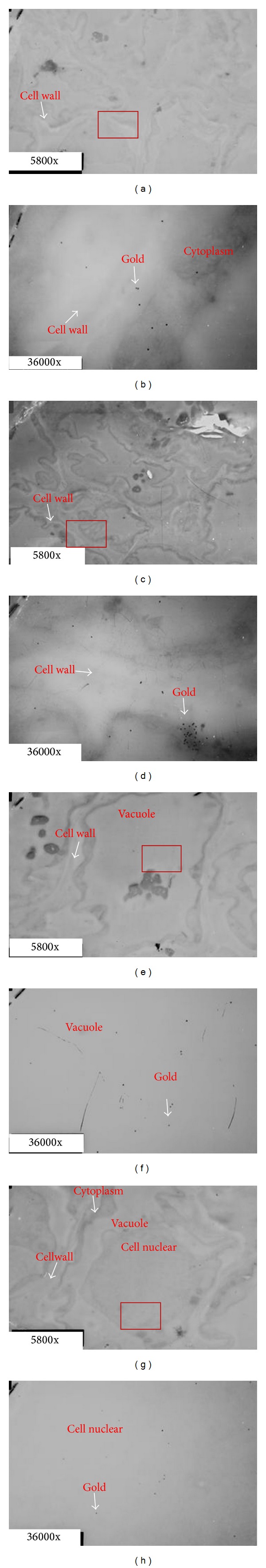
Transmission electron micrographs of tomato pedicel and localization of polygalacturonase (PG) in abscission zone cells by immunogold labeling (proximal end, 24 h). (a) and (b): cortex; (c) and (d): vascular region; (e) and (f): vacuole; (g) and (h): cell nucleus; (b), (d), (f), and (h) are enlarged photos for the rectangles region in (a), (c), (e), and (g), respectively.

**Figure 8:**
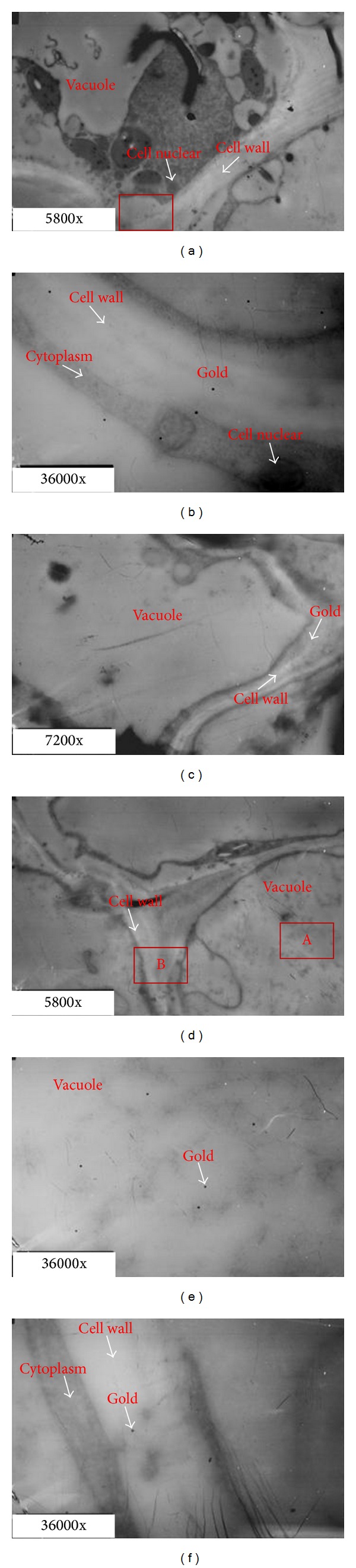
Transmission electron micrographs of tomato pedicel and localization of polygalacturonase (PG) in abscission zone cells by immunogold labeling (Distal end, 24 h). (a), (b), and (c): cortex; (d), (e), and (f): vascular region; (b) is enlarged photo for the rectangles region in (a); (e) and (f) are enlarged photos for the rectangles region of A and B in (d), respectively.

After 24 h of ethylene treatment, the abscission zone tissue was completely broken down into two parts: proximal side (Figure 7) and distal side (Figure 8). On the surface of the abscission zone, cell structure completely decomposed and showed a diffusion-like distribution. The cell wall of inside cells degraded, and most of the cells were free. The cells with severe degradation lost inclusions (Figure 7). At this point, a large number of gold particles were distributed at the proximal side of the abscission zone. Gold particles also gathered in some regions. These findings indicated that the existence of a large number of polygalacturonic acid enzymes in the abscission tissue was crucial in the degradation of the cell wall and other substances. In addition, a large number of gold particles were observed on the distal side of the abscission zone (Figure 8), indicating that the polygalacturonic acid enzyme was expressed in all organizations, including the proximal and distal sides of the abscission zone.

## 4. Discussion

The results clearly demonstrated the distribution of PG in the tomato pedicel abscission zone after immunogold staining. Since Faulk and Taylor [15] first used immunogold staining to demonstrate antigens by electron microscopy, various modifications have helped improve the sensitivity of the method, which has now become a mature technology in immunohistochemistry [16–18]. Cellular localization of the endo-PG protein in* Arabidopsis *siliques was determined by Sander et al. [19]. They showed that PG is confined to the dehiscence zone, which is similar to the abscission zone. In the present study, we used two dimensions of colloidal gold (10 and 15 nm). However, 10 nm colloidal gold particles are difficult to detect within the scope of electron microscopy (data not shown). Hence, we recommend the use of 15 nm colloidal gold particles in future research.

The tomato pedicel abscission zone is ideal for observing abscission. This zone contains several layers of small and regular cells with dense cytoplasm. Its morphology can also be easily distinguished from other tissues. Thus, samples can be cut by hand, and the exact location of the abscission zone can be found easily through electron microscopy.

The activity of PG is correlated with abscission in different plant varieties; PG is also an indicator of the progress of abscission [8, 10, 20–25]. At the gene level, Kalaitzis et al. [10] pointed out that three PGs (TAPG1, TAPG2, and TAPG4) are expressed in the leaf and flower abscission zones as well as in the pistils of fully opened flowers. However, TAPG4 mRNA is detected much earlier than TAPGl and TAPGZ mRNA during both leaf and flower abscission. González-Carranza et al. [25] detected PG gene expression in cells at the base of the anther filament (stamen), petals, and sepals in the flowers of transgenic* Arabidopsis*. At the enzyme level, Robert [26] revealed that the related PG activity is only limited to the distal part of the abscission zone. However, Wang et al. [14] indicated that PG activity is significantly increased at the distal and proximal ends of the tomato pedicel abscission zone during separation. In the present paper, PG initially increased in the parenchyma cells in the cortex and vascular tissues in the abscission zone, along the side of the vascular tissue extending to the distal and proximal ends, and then lateral from outside to the central region of the abscission zone. These results suggested that PG was closely related to abscission. At first, PG was only observed in the cell wall and nearby region. However, it also appeared in the nucleus during separation, which showed the* de novo* biosynthesis of PG in abscission.

In abscission, PG is responsible for the degradation of the middle lamella and for the loosening of the primary cell wall. Recently, alterations in the chemical composition of the cell wall during abscission have indicated that the deposition of certain substances, such as xyloglucan and galactan for floral abscission and lignin for fruit, occurs specifically at the abscission zone [27]. Thompson and Osborne [28] demonstrated that the stele is the source of abscission and speculated that an oligosaccharide released from the cell walls of the stele may act as the signal for cortical cells to begin cell separation at the tomato pedicel abscission zone. Kalaitzis et al. [10] then believed that the early expression of TAPG4 is involved in a similar signaling process in tomato. In this trial, the increase in PG was first found only at the cell wall region of the cortical and vascular regions in the abscission zone and then along the vascular tissue extending to the distal and proximal ends. These results suggested the function of PG in producing the abscission signal and degrading the middle lamella.
